# The Diagnostic Accuracy of a Fecal Immunochemical Test in Detecting Colorectal Cancer and Advanced Precancerous Colorectal Neoplasia in Patients with Iron Deficiency: A Protocol for Systematic Review and Meta-Analysis

**DOI:** 10.1155/2023/5982580

**Published:** 2023-12-08

**Authors:** Jennifer Pham, Geraldine Laven-Law, Jean M. Winter, Molla M. Wassie, Charles Cock, Erin L. Symonds

**Affiliations:** ^1^Department of Medicine, College of Medicine and Public Health, Flinders University, Bedford Park, SA 5042, Australia; ^2^Cancer Research, Flinders Health and Medical Research Institute, Flinders University, Bedford Park, SA 5042, Australia; ^3^Department of Gastroenterology, Flinders Medical Centre, Bedford Park, SA 5042, Australia

## Abstract

*Background*. Iron deficiency (ID) is a common micronutrient deficiency and the leading cause of anemia worldwide. ID can be caused by chronic occult blood loss from colorectal neoplasia including colorectal cancer (CRC) and advanced precancerous colorectal lesions. Current guidelines recommend colonoscopy in both men and postmenopausal women presenting with ID anemia (IDA). However, there is controversy on the investigation of patients presenting with a lower risk of CRC including younger women with ID and those with nonanemic ID (NAID). There is a need for a triaging tool to identify which ID patients may benefit from colonoscopy. The fecal immunochemical test (FIT) is sensitive for CRC screening in an asymptomatic population, but its role in ID patients is unclear. The aim of this study is to conduct a systematic review to determine the diagnostic accuracy of FIT for detecting CRC and advanced precancerous neoplasia in individuals presenting with ID with or without anemia. *Methods and Analysis*. This protocol conforms with the Preferred Reporting Items for Systematic Reviews and Meta-Analyses Protocols and *Cochrane Handbook for Systematic Reviews of Diagnostic Test Accuracy*. A comprehensive search of the MEDLINE, Embase, and Web of Science databases will be undertaken for studies published after 2010 which involve patients with ID, who completed a FIT in the 6 months prior to colonoscopy, with FIT sensitivity and specificity calculated against the reference standard colonoscopy. The search will be limited to studies conducted after 2010 to reduce variability in colonoscopy quality. Risk of bias assessment will be conducted using the Quality Assessment of Diagnostic Accuracy Studies version 2. FIT sensitivity and specificity will be the primary measure of diagnostic accuracy, and data will be analysed using a random effects meta-analysis. *Discussion*. This review and meta-analysis will be the first to systematically explore the value of the FIT as a triaging tool for patients with ID. This trial is registered with CRD42022367162.

## 1. Introduction

Iron deficiency (ID) is a highly prevalent micronutrient deficiency and is the leading cause of anemia globally [1, 2]. While the World Health Organization defines ID as <15 *μ*g/L (15 ng/mL, 0.0337 nmol/L, and 1.5 *μ*g/dL) of serum ferritin in adults [3], <30 *μ*g/L (30 ng/mL, 0.0674 nmol/L, and 3 *μ*g/dL) is commonly used as a cutoff for clinical diagnosis [4, 5]. ID anemia (IDA) occurs when ID is severe enough to affect hemoglobin (Hb) synthesis and is defined as a Hb concentration below 130 g/L (13 g/dL, 130 mg/mL) in men, 120 g/L (12 g/dL, 120 mg/mL) in nonpregnant women, and 110 g/L (11 g/dL, 110 mg/mL) in pregnant women [6].

One of the causes of ID is chronic occult blood loss from colorectal neoplasia [6]. Colorectal cancer (CRC) is the third most commonly diagnosed cancer and is the second leading cause of cancer-related death globally [7]. CRC develops from two types of precancerous neoplasia: adenomatous polyps and sessile serrated lesions [8]. Polyps that are at higher risk of turning malignant include advanced adenomas (adenomas with a diameter of 1 cm or greater and/or villous features and/or high-grade dysplasia) [8] and advanced serrated lesions (sessile serrated lesions with a diameter of 1 cm or greater and/or with dysplasia, or all traditional serrated adenomas) [9]. Such larger lesions are also more likely to bleed [10–12]. Early detection of precancerous neoplasia allows an opportunity to remove growing lesions before they develop into CRC, thereby reducing CRC incidence and mortality [13].

Current guidelines recommend colonoscopy in men and postmenopausal women presenting with IDA to exclude CRC and precancerous neoplasia [14]; however, the investigation of lower risk patients including nonanemic ID (NAID) and younger premenopausal women remains a clinical challenge [15–17]. The cost of colonoscopy in low-risk ID patients may not outweigh the benefits: the procedure can be costly and inconvenient and has long wait times with risks of bleeding, bowel perforation, and death [18]. The risk of colorectal neoplasia varies, and while up to approximately 8.9% of patients may have a malignancy identified at colonoscopy [17], others with ID are at much lower risk (<1%) of CRC [19]. In this low-risk group, the risks and costs associated with colonoscopy may therefore outweigh the benefits, and resources may be better directed to those at higher risk.

The fecal immunochemical test (FIT) has been used in primary care in Australia, Spain, and the UK to help determine which patients presenting with low-risk CRC symptoms should be referred for colonoscopy [20]. The FIT detects occult blood using antibodies that bind to the globin moiety of Hb in a provided fecal sample [21]. The quantitative concentration of occult Hb within the FIT is measured, allowing different positivity thresholds to be used [21]. Currently, only 3 countries have adopted the use of FIT in primary care, as there is still a limited body of evidence to support the use of FIT as a triaging tool in symptomatic patients [20]. However, a systematic review has shown that FIT can have a sensitivity of up to 90% and specificity of 87% in symptomatic patients [22]. There is no consensus on the use of FIT in patients with IDA [14] and very limited evidence on the use of FIT in NAID. Additionally, the optimal FIT threshold for detecting CRC and precancerous neoplasia in symptomatic and ID patients remains unclear. Therefore, this project is aimed at evaluating the existing evidence on the diagnostic accuracy, inclusive of the sensitivity and specificity, of FIT for detecting CRC and precancerous colorectal neoplasia in patients with ID, with or without anemia, compared to the reference standard colonoscopy and further determining the optimal FIT positivity threshold which can be used to triage patients with ID ahead of colonoscopy.

## 2. Methods and Analysis

### 2.1. Study Registration

The protocol was developed following the Preferred Reporting Items for Systematic Reviews and Meta-Analyses (PRISMA) 2020 guidelines and the *Cochrane Handbook for Systematic Reviews of Diagnostic Test Accuracy*. The protocol is registered with PROSPERO and is accessible from https://www.crd.york.ac.uk/prospero/display_record.php?ID=CRD42022367162.

### 2.2. Eligibility Criteria

#### 2.2.1. Population

The study participants of interest will be adult patients (aged ≥18 years) diagnosed with ID, with or without anemia. Individuals with a known coexisting cancer and/or a genetic predisposition for CRC (e.g., Lynch syndrome and familial adenomatous polyposis) will be excluded due to an above-average risk for CRC. Where known, individuals who have been diagnosed with inflammatory bowel disease prior to colonoscopy will be excluded, as this condition can also lead to occult blood loss and a false-positive FIT. Individuals with known hematological conditions will be excluded due the conditions' effect on blood results and iron studies. Individuals with known coeliac disease will similarly be excluded due to potential malabsorption of iron.

#### 2.2.2. Interventions

This review will examine the diagnostic accuracy of the FIT for the detection of advanced colorectal neoplasia in individuals with ID with or without anemia. FIT must be completed within 6 months prior to diagnostic colonoscopy, and only studies using quantitative FITs in which Hb thresholds are reported will be included. All other types of fecal occult blood test, including guaiac-based tests, will be excluded.

#### 2.2.3. Comparators

Colonoscopy is considered the gold standard test for identifying both precancerous neoplasia and CRC [23]. As such, the diagnostic accuracy of FIT will be compared to colonoscopy. All other endoscopic methods as sigmoidoscopy and computed tomography (CT) colonography will be excluded.

#### 2.2.4. Outcomes

The primary outcome will be the diagnostic accuracy of quantitative FIT compared to the reference standard colonoscopy for the identification of advanced colorectal neoplasia in individuals with ID with or without anemia. The sensitivity and specificity of FIT will be determined separately for diagnosis of CRC, advanced colorectal neoplasia (inclusive of CRC and any advanced precancerous colorectal neoplasia), advanced conventional adenomas, advanced serrated lesions, and advanced precancerous neoplasia (inclusive of advanced conventional adenomas and advanced serrated lesions). Only studies that have sensitivity and specificity data of FIT for detecting advanced colorectal neoplasia will be included. Other measures of diagnostic accuracy, including positive predictive values, negative predictive values, and odds ratios, will be included where available.

The secondary outcomes will include the following:

1. The optimal FIT positivity threshold to refer individuals with ID for colonoscopy
2. The diagnostic accuracy of FIT to identify precancerous neoplasia/or CRC for different population groups (e.g., different age groups, males, and females)
3. The diagnostic accuracy of FIT to identify precancerous neoplasia and/or CRC for individuals with IDA compared to NAID
4. The diagnostic accuracy of FIT for different CRC types (e.g., location, staging, and histological subtype where available)
5. The diagnostic accuracy of FIT for precancerous neoplasia compared to CRC

The optimal threshold for FIT will be determined using a summary receiver operating characteristic (SROC) curve. The sensitivity and specificity of FIT and other measures of diagnostic accuracy including positive predictive values, negative predictive values, and odds ratios will be included where available for different colonoscopy outcomes and study demographics.

#### 2.2.5. Studies

Colonoscopy quality has improved significantly over time due to technological advances. The high-resolution colonoscopy first introduced in 2005 has over the years replaced traditional colonoscopy methods due to its improved visualisation and neoplasia detection rate and is currently the standard of care [24, 25]. A study analysing the colonoscopy quality from 2000 to 2014 found that colonoscopic bowel preparation and polyp identification improved after 2010 in both average risk screening and surveillance colonoscopies [26]. As such, only studies that conducted data collection after 2010 will be included in the review to minimise variability due to colonoscopy quality. Studies that commenced data collection before 2010, even if it continued beyond 2010 (e.g., 2008-2012), will be excluded. In addition, by 2010, FIT was found to be more sensitive in detecting colorectal neoplasia and resulted in the higher participation rates compared to guaiac fecal occult blood tests and has been widely used in CRC screening programs globally [27, 28]. Cohort studies, cross-sectional studies, case-control studies, randomised control trials and clinical trials will be included. Reviews and case studies will be excluded. Summary of the inclusion and exclusion criteria is provided in Table 1.

### 2.3. Information Sources

Studies will be identified through a literature search from inception to search date in the MEDLINE, Web of Science, and Embase databases. Further studies will be identified from reference lists of eligible studies and review articles. The search will be limited to studies conducted after 2010. The search will be rerun before final analysis to include recently published articles. Only published articles will be included. This will include published conference abstracts that have sufficient data.

### 2.4. Search Strategy

The search strategy will be developed by two reviewers (JP and MMW) with the support of a research librarian. All other reviewers will provide feedback on the search strategy. The master search will be developed on MEDLINE using appropriate Medical Subject Headings (MeSH) keywords as described in Supplementary Table 1. MeSH keywords will be adjusted as necessary for the remaining two databases. The final search will be done all on the same day and recorded.

### 2.5. Data Management

Studies and their citation and abstract will be downloaded into EndNote V.20 (Clarivate Analytics) and then uploaded to Covidence, a systematic review management software. The inclusion and exclusion criteria will be uploaded on Covidence to assist reviewers in screening.

### 2.6. Study Selection

#### 2.6.1. Title and Abstract Screening

The screening will be done according to PRISMA 2020 guidelines and through using the Covidence program, a systematic review management system [29]. Prior to screening, the inclusion and exclusion criteria for studies will be discussed amongst all reviewers to develop a clear guideline. The first phase of screening will be on article titles and abstracts of all studies identified from the search. Studies will be divided amongst the 5 reviewers with each study requiring assessment by 2 independent reviewers. Decisions by individual reviewers will be recorded on Covidence. Covidence ensures that the decisions made by reviewers are blinded to other reviewers. Reasons for exclusion in this phase of screening will not be recorded. Studies will be excluded if they are irrelevant to the research question. Studies that mention keywords relevant to the research question or needing further evaluation of full text to determine relevance will be included. The Covidence tool will flag disagreements in the screening. The disagreements will be resolved via discussion with all members of the review team.

#### 2.6.2. Full-Text Screening

Once all studies have been assessed and conflicts resolved, the remaining articles that pass the title and abstract screening will undergo full-text screening. Similarly, the studies will be divided amongst the reviewers with each study requiring a full-text assessment by 2 independent reviewers. The screening decisions will be recorded on Covidence and will be kept blinded. Reviewers will review the full-text articles and use the predefined inclusion and exclusion criteria to determine eligibility of studies. Reviewers will keep a record of their reasons for excluding or including studies that they have assessed. The reasons for exclusion in the full-text screening will be recorded and included in the final PRISMA 2020 flow diagram. Disagreements regarding inclusion, as well as reason for exclusion of full studies will be flagged by Covidence and will be discussed with all members of the review team until a consensus is achieved. Studies may have multiple different reasons for being excluded. The review team will discuss this and develop a hierarchy order of different reasons of exclusion to be applied to studies that have multiple reasons for exclusion. Studies that pass the full-text screening will move on to data extraction.

#### 2.6.3. Assessing the Reliability between Reviewers

The interrater reliability (IRR) will be calculated to determine the reliability of decisions made by different reviewers. Cohen's kappa can be used to assess IRR. Cohen's kappa value will be interpreted as values 0-0.20 indicating no agreement, 0.21–0.39 as minimal agreement, 0.40-0.59 as weak agreement, 0.60-0.79 as moderate agreement, 0.80-0.90 as strong agreement, and above 0.90 as almost perfect agreement [30].

### 2.7. Data Extraction and Collection

Data will be extracted from eligible studies that have passed the full-text screening by two independent reviewers on separate Microsoft Excel sheets using a premade template. Data that will be extracted will include background information including authors, year of publication, year the study was conducted, aim of study, country of study, inclusion and exclusion criteria, study setting, population demographics (age, sex), and recruitment strategy. Information about the methodology of included studies that will be extracted will include the study type, definition of ID and anemia including ferritin and hemoglobin thresholds, definition of colorectal precancerous neoplasia, FIT details (brand/company, number of samples, FIT instructions, and FIT positivity thresholds), colonoscopy procedure (procedure instructions, quality, and outcomes), and the time between FIT and colonoscopy. Results that will be extracted are sample size, prevalence of CRC and precancerous neoplasia, sensitivity and specificity of FIT, and raw data including true positives, true negatives, false positives, and false negatives if available, and additional measures of diagnostic accuracy such as positive predictive value and negative predictive values may be calculated from data where available. The extracted data will then be compared for discrepancies, and disagreements will be resolved by a third reviewer.

### 2.8. Dealing with Missing Data

If articles are missing data, or further clarification is required, the corresponding author will be contacted by email, telephone, or other means to request missing data or additional information. If a study is found to be nearly eligible for inclusion (e.g., some patients in the study underwent CT colonography and others underwent a colonoscopy, but all other inclusion and exclusion criteria are met), the corresponding authors will be contacted to extract data subsets matching the inclusion criteria of this systematic review. If authors do not respond within one month, a second attempt to contact them will be made. If sufficient information remains unobtainable, we will try and use the available coefficients to calculate the data. If available information is insufficient, and the corresponding author does not respond to the data request within one month of the second attempt at contact, the study will be excluded. The impact of missing data on the results will be explained in the final review.

### 2.9. Data Items

A summary of extracted data is provided in Table 2.

### 2.10. Risk of Bias

The methodological quality of each eligible study will be assessed using Quality Assessment of Diagnostic Accuracy Studies, 2^nd^ edition (QUADAS-2) [31], by evaluating risk of bias over four key domains: participant selection, index test, reference standing, and flow and timing [31]. This tool also assesses whether the eligible studies are applicable to answer the review question and involves the use of signalling questions to assess bias over each domain. Prior to conducting the risk of bias assessment, reviewers will work together to develop a clear guideline on how to answer each of the signalling questions and how to use these questions to assess bias and applicability in each domain. All eligible studies will be subject to risk of bias assessment by a minimum of two independent reviewers. Discrepancies in the methodological quality of eligible studies between reviewers will be resolved through arbitration with all members of the review team.

### 2.11. Data Synthesis

The primary measure of diagnostic accuracy of the FIT for detecting CRC and colorectal precancerous neoplasia or all neoplasia (CRC and precancerous lesions) combined will be assessed using sensitivity and specificity measurements, inclusive of 95% confidence intervals. Other measures of diagnostic accuracy, including positive predictive values, negative predictive values, and odds ratios, will also be calculated wherever possible. Sensitivity and specificity of individual studies will be combined using a random effects meta-analysis. True positives, true negatives, false positives, and false negatives will be required for the random effects model. The optimal positivity threshold for the FIT will be determined for different FIT thresholds using an SROC curve. Area under the curve (AUC) will be used to assess the diagnostic accuracy of FIT. Heterogeneity will be assessed using chi-squared and *I*-squared tests as appropriate. The *I*-squared test interpretation will follow Cochrane guidelines [32]. Where possible, subgroup analysis will be performed to compare the diagnostic accuracy of FIT in different groups such as different demographic groups and comparing individuals with IDA compared to NAID.

### 2.12. Ethics and Dissemination of Results

This study is being conducted using systematic review and meta-analysis methods. Existing published trial data will be used throughout this study. Individual patient data will not be used, and as such, there are no ethical considerations associated with this protocol. The results will be published in a peer-reviewed journal and presented at conferences.

## 3. Discussion

A small number of countries, including Australia, the UK, and Spain, have recommended FIT to investigate patients with low-risk CRC symptoms [20]. However, there is limited evidence to support these recommendations in symptomatic patients. Further, the value of the FIT for investigating ID with or without anemia remains unclear. The aim of this review is to determine the diagnostic accuracy of FIT for detecting CRC and precancerous neoplasia in patients presenting with ID. This will provide guidance to clinicians on whether FIT is an appropriate triaging tool for determining the need for diagnostic colonoscopy in individuals with ID. Additionally, this review may be able to provide an optimal FIT Hb threshold that can be used in the investigation of ID patients. This review will evaluate the current evidence on both IDA and NAID. Since there is limited evidence on NAID, this research will provide more guidance on managing NAID and identify gaps that need further research. Overall, the results of this review will provide more evidence and clinical guidance in managing ID patients with or without anemia in primary care.

## Figures and Tables

**Table 1:** Summary of inclusion and exclusion criteria.

	Inclusion criteria	Exclusion criteria
Population	(i) 18 years and older (ii) Any sex (iii) Diagnosed with ID, with or without anemia	(i) Known coexisting cancer of any type (ii) Known genetic predisposition for CRC including Lynch syndrome and familial adenomatous polyposis (iii) Known inflammatory bowel disease (iv) Known hematological diseases (v) Known coeliac disease

Intervention	(i) Quantitative FIT	(i) Guaiac-based fecal test (ii) Nonquantitative FIT

Comparison	(i) Colonoscopy done within 6 months after FIT	(i) Other screening options including sigmoidoscopy and CT colonography

Study type	(i) Cohort studies (ii) Cross-sectional studies (iii) Case-control studies (iv) Clinical trials (v) Randomised controlled trials	(i) Reviews (ii) Case studies (iii) Animal models (iv) In vitro studies

**Table 2:** Summary of items to be extracted from included studies.

Information area	Data to be extracted
Background	Authors
	Year of publication
	Year of study
	Aim of study
	Country of study
	Inclusion and exclusion criteria
	Study setting
	Population demographics
	Recruitment strategy

Methodology	Study type
	Definition of ID and anemia
	Definition for colorectal precancerous neoplasia
	FIT information including brand/company, number of samples, how FIT was performed, FIT positivity thresholds
	Colonoscopy procedure including quality and completeness
	Colonoscopy outcomes
	Time between FIT and colonoscopy

Results	Sample size
	Prevalence of CRC and precancerous neoplasia
	Sensitivity of FIT
	Specificity of FIT
	Raw data including true positives, true negatives, false positives, false negatives if available
	Other diagnostic accuracy data including positive predictive value, negative predictive value, odds ratio if available

## Data Availability

The data and materials are available from the corresponding author upon reasonable request.

## Abbreviations

AUC: Area under the curve
CRC: Colorectal cancer
CT: Computed tomography
FIT: Fecal immunochemical test
ID: Iron deficiency
IDA: Iron deficiency anemia
IRR: Inter-rater reliability
Hb: Hemoglobin
MeSH: Medical subject headings
NAID: Nonanemic iron deficiency
PRISMA: Preferred Reporting Items for Systematic Reviews and Meta-Analyses
QUADAS-2: Quality Assessment of Diagnostic Accuracy Studies
SROC: Summary receiver operating characteristic.

## Supplementary Materials

Supplementary Materials Supplementary Table 1: keywords and MeSH (Medical Subject Headings) used for different databases in the systematic search.
