# Supplementary material for: The Diagnostic Accuracy of a Fecal Immunochemical Test in Detecting Colorectal Cancer and Advanced Precancerous Colorectal Neoplasia in Patients with Iron Deficiency: A Protocol for Systematic Review and Meta-Analysis
Source: Gastroenterol Res Pract. 2023 Dec 8;2023:5982580. doi: 10.1155/2023/5982580 (PMC10723928; doi:10.1155/2023/5982580)
Supplement: Supplementary Materials — Supplementary Table 1: keywords and MeSH (Medical Subject Headings) used for different databases in the systematic search. [file 5982580.f1.pdf]

Supplementary table 1 – Keywords and MeSH used for different databases in the systematic search

| | | Master search | MEDLINE | Web of science | EMBASE |
|---|---|---|---|---|---|
| Concept 1 – Iron deficiency | Keywords | Iron or "iron deficien*" or IDA | (Iron or "iron deficien*" or IDA).mp. | Iron or "iron deficien*" or IDA | (Iron or "iron deficien*" or IDA).mp |
| | MeSH | exp Iron Deficiencies/ | exp Iron Deficiencies/ | N/A | exp Iron Deficiency/ |
| Concept 2 – Faecal immunochemical test | Keywords | "faecal immuno* test*" or "fecal immuno* test*" or FIT or FITs or qFIT or "faecal occult" or "fecal occult" or "stool occult" or FOB* or iFOB* or "immuno* fecal" or "immuno* faecal" or "immuno* stool" or "occult blood" | ("f?ecal immuno* test*" or FIT or FITs or qFIT or "f?ecal occult" or "stool occult" or FOB* or iFOB* or "immuno* f?ecal" or "immuno* stool" or "occult blood").mp. | "f*ecal immuno* test*" or FIT or FITs or qFIT or "f*ecal occult" or "stool occult" or FOB* or iFOB* or "immuno* f*ecal" or "immuno* stool" or "occult blood"). | ("f?ecal immuno* test*" or FIT or FITs or qFIT or "f?ecal occult" or "stool occult" or FOB* or iFOB* or "immuno* f?ecal" or "immuno* stool" or "occult blood").mp |
| | MeSH | exp Occult Blood/ | exp Occult Blood/ | N/A | exp occult blood test/ or exp occult blood/ |
| Concept 3 – Colorectal cancer | Keywords | ((Colo* or bowel or rect* or caec* or cec* or sigmoid* or intestin* or gastrointestin* or GI) and (adeno* or serrated or polyp* or cancer* or carcinoma* or neoplas* or malignan* or lesion* or tumour* or tumor*)) or CRC. | (((Colo* or bowel or rect* or caec* or cec* or sigmoid* or intestin* or gastrointestin* or GI) and (adeno* or serrated or polyp* or cancer* or carcinoma* or neoplas* or malignan* or lesion* or tumour* or tumor*)) or CRC).mp. | ((Colo* or bowel or rect* or caec* or cec* or sigmoid* or intestin* or gastrointestin* or GI) and (adeno* or serrated or polyp* or cancer* or carcinoma* or neoplas* or malignan* or lesion* or tumour* or tumor*)) or CRC. | (((Colo* or bowel or rect* or caec* or cec* or sigmoid* or intestin* or gastrointestin* or GI) and (adeno* or serrated or polyp* or cancer* or carcinoma* or neoplas* or malignan* or lesion* or tumour* or tumor*)) or CRC).mp. |
| | MeSH | exp Intestinal Polyps/<br>exp intestinal neoplasm/<br>exp neoplasm/ | exp Intestinal Polyps/<br>exp intestinal neoplasm/<br>exp neoplasm/ | N/A | exp intestine polyp/<br>exp intestine tumor/<br>exp neoplasm/ |
